# Supplementary material for: Understanding Molecular Basis of PTPN11-Related Diseases
Source: ArXiv. 2025 Nov 14:arXiv:2511.11860v1. Preprint. [Version 1] (PMC12754711)
Supplement: Supplement 1 [file NIHPP2511.11860v1-supplement-1.pdf]

Supplementary materials

variant	disease	MutPred2 score
T2I	NS	0.648
T42A	NS	0.502
I56V	NS	0.629
N58D	NS	0.863
N58K	NS	0.902
T59A	NS	0.666
G60A	NS	0.875
D61Y	NS	0.932
D61G	NS	0.903
D61N	NS	0.821
Y62C	NS	0.848
Y62D	NS	0.944
Y63C	NS	0.916
E69Q	NS	0.672
F71I	NS	0.891
F71L	NS	0.884
A72V	NS	0.505
A72S	NS	0.380
A72G	NS	0.523
T73I	NS	0.869
E76A	NS	0.876
E76D	NS	0.766
Q79R	NS	0.753
Q79P	NS	0.930

D106A	NS	0.871
E110A	NS	0.820
E139D	NS	0.811
Q256R	NS	0.646
L261F	NS	0.499
L261H	NS	0.776
L262F	NS	0.512
L262R	NS	0.844
R265Q	NS	0.622
Y279C	NS	0.948
I282M	NS	0.850
I282V	NS	0.629
F285S	NS	0.961
F285L	NS	0.942
N308D	NS	0.771
N308S	NS	0.728
I309V	NS	0.257
T411M	NS	0.322
P491S	NS	0.808
R501L	NS	0.937
R501K	NS	0.889
S502T	NS	0.746
G503V	NS	0.925
G503R	NS	0.925
M504V	NS	0.910
Q506R	NS	0.908

Q510R	NS	0.897
Q510E	NS	0.890
L560F	NS	0.237
Y279C	LS	0.948
Y279S	LS	0.955
A461T	LS	0.765
G464A	LS	0.934
T468M	LS	0.926
R498L	LS	0.948
R498W	LS	0.940
Q506P	LS	0.960
Q510E	LS	0.890
Q510P	LS	0.952
D61H	JMML	0.900
D61Y	JMML	0.932
D61V	JMML	0.893
Y62D	JMML	0.944
E69V	JMML	0.817
E69K	JMML	0.761
A72V	JMML	0.505
A72T	JMML	0.483
T73I	JMML	0.869
E76K	JMML	0.888
E76G	JMML	0.876
E76A	JMML	0.876
E76V	JMML	0.891

E76Q	JMML	0.800
R265Q	JMML	0.622
R289G	JMML	0.952
S502L	JMML	0.903
S502T	JMML	0.746
G503A	JMML	0.860
G503R	JMML	0.925
G503V	JMML	0.925
Q506P	JMML	0.960

Supplementary Table 1. List of PTPN11 variants along with associated diseases and pathogenicity scores predicted by Mutpred2.

Residue 1 (Chain)	Residue 2 (Chain)	(Å)
36 (N)	163 (C)	7.59
36 (N)	161 (C)	7.55
37 (N)	163 (C)	7.34
37 (N)	161 (C)	7.26
47 (N)	449 (P)	7.24
46 (N)	316 (P)	7.07
81 (N)	160 (C)	7.04
47 (N)	448 (P)	7.04
47 (N)	408 (P)	7.04
47 (N)	409 (P)	7.01

Supplementary Table 2. Top 10 residue pairs exhibiting the largest distance changes in the difference matrix ∆ induced by the D61N variant; N: N-SH2; C: C-SH2; P: PTP.
